# Artificial intelligence in hepatocellular carcinoma screening: applications and challenges

**DOI:** 10.3389/fmed.2025.1713887

**Published:** 2026-01-16

**Authors:** Jian-Xu Rao, Ying Li, Kai Leng

**Affiliations:** 1 Department of Hepatopancreatobiliary Surgery, The First People’s Hospital of Zunyi (The Third Affiliated Hospital of Zunyi Medical University), Zunyi, Guizhou, China; 2 Department of Gastroenterology and Hepatology, West China Hospital, Sichuan University, Chengdu, Sichuan, China

**Keywords:** artificial intelligence, challenges, deep learning, future perspectives, hepatocellular carcinoma screening, machine learning, applications

## Abstract

Hepatocellular carcinoma (HCC) is the predominant histological subtype of primary liver cancer, with a 5-year survival rate of approximately 18%. Early detection of HCC is critical for guiding treatment selection and improving patient survival outcomes. The effectiveness of conventional screening methods is decreased due to their inherent limitations and individual variability. Artificial intelligence (AI) has advanced rapidly in medical practice and has played a significant role in increasing the early detection rates of HCC by replacing manual tasks and accessing hidden information in routinely available clinical data. However, numerous challenges, such as ethical concerns, model instability, and generalizability, must be overcome before their full clinical implementation. This article reviews recent studies that describe AI-based models for the early diagnosis of HCC, focuses on the current applications and persistent challenges of AI in HCC screening and discusses its perspectives. We aim to provide a critical evaluation of the potential of AI for enhancing early HCC detection and improving patient prognosis.

## 1 Introduction

Primary liver cancer ranks as the sixth most common malignancy and the third leading cause of cancer-related mortality worldwide, representing a significant global health burden (1, 2). It primarily includes hepatocellular carcinoma (HCC), intrahepatic cholangiocarcinoma (ICC), and combined hepatocellular-cholangiocarcinoma (HCC-ICC). HCC accounts for approximately 90% of all primary liver cancers and is associated with a 5-year survival rate of approximately 18% (3, 4). Early detection is critical for both therapeutic decision-making and patient prognosis (5).

Screening is important for the early detection of HCC, but its effectiveness is limited by multiple barriers (6, 7). Patient-related obstacles include misconceptions about screening, financial constraints, limited healthcare access, difficulties in scheduling appointments, long intervals between visits and ultrasounds, and transportation issues (8–10). Provider-related barriers include insufficient knowledge of guidelines, failure to identify at-risk patients, time constraints, and resource limitations (11, 12). These factors collectively undermine suboptimal screening adherence. Furthermore, current HCC screening strategies are limited by shifting etiologies, notably, the rise of metabolic dysfunction-associated steatotic liver disease (MASLD) and alcohol-related liver disease (ALD) against a background of persistent viral hepatitis (13, 14). This epidemiological shift, characterized by a large at-risk population, a lower incidence of HCC in non-cirrhotic individuals, and ongoing cost-effectiveness concerns, substantially complicates the justification for widespread screening (15, 16). The screening modalities used for HCC patients also face significant limitations. However, tumor biomarkers for accurate early detection are still lacking. The available data show that biomarkers are suboptimal in terms of cost-effectiveness for routine surveillance of early HCC (6). Ultrasound has several inherent limitations, including operator dependence, suboptimal visualization in obese patients, and the risks associated with false-positive or indeterminate findings, potentially compromising its efficacy (17). Although contrast-enhanced computed tomography (CT) provides high diagnostic accuracy, its routine use is constrained by radiation exposure. Magnetic resonance imaging (MRI) demonstrates superior sensitivity for small lesions but is less feasible for population-level screening because of longer scan times, high cost, and complex interpretation.

**FIGURE 1:**
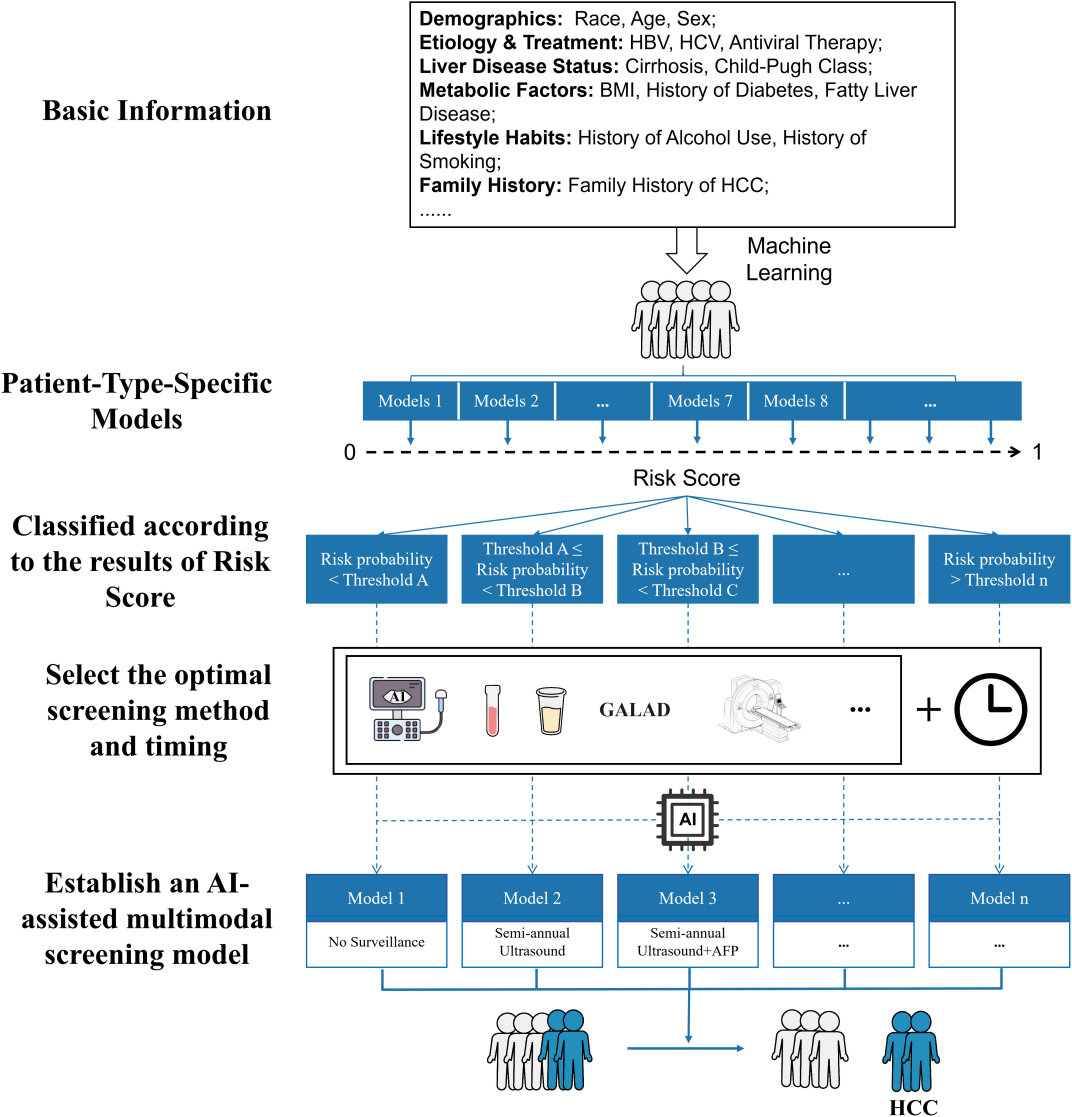
Flowchart of an AI-assisted precision surveillance strategy for HCC. The framework integrates readily available patient data—including demographics, etiology, liver disease status, and metabolic factors—to generate a personalized risk score. Based on this score, patients are stratified into distinct risk categories. For each category, a tailored screening strategy (e.g., no surveillance, semi-annual ultrasound with or without AFP…) is selected and implemented via a dedicated AI-assisted model, enabling precision in early detection. HBV, hepatitis B virus; HCV, hepatitis C virus; BMI, body mass index; HCC, hepatocellular carcinoma; AI, artificial intelligence; US, ultrasound; AFP, Alpha-fetoprotein.

Artificial intelligence (AI) is defined as a broad concept involving the use of computer systems to perform complex functions that could require human intelligence, quantifying uncertainty, probabilistic reasoning, problem-solving behaviors and knowledge representation. The field of AI encompasses various methodologies and technologies, such as machine learning and its subset deep learning, which employ algorithms including artificial neural networks (ANNs) and convolutional neural networks (CNNs) to enable applications in areas like image processing and computer vision (18). The implementation of AI-powered diagnostic technologies that integrate multifactorial analysis is essential to achieve precise diagnostics and advance personalized medicine. Given the heterogeneity of HCC, AI can extract critical information and leverage massive amounts of multiparametric data (Figure 1). The application of AI in HCC screening has significantly improved the early detection rate of HCC.

In the management of HCC, it is crucial to differentiate between the application of AI for screening and diagnostic classification. Screening involves the use of AI tools in asymptomatic, high-risk populations with the primary objective of detecting potential HCC at an early stage. It aims to achieve high sensitivity and minimize the rate of missed diagnoses (19). In contrast, diagnostic classification entails the use of AI models to assist in the characterization of identified focal liver lesions (FLLs). These models aim to differentiate between benign and malignant nodules or to further classify specific HCC subtypes. Consequently, this application prioritizes high specificity and overall diagnostic accuracy (20, 21). Studies have demonstrated that developed AI models exhibit excellent performance in the differential diagnosis of HCC, achieving area under the curve (AUC) values exceeding 0.90. Furthermore, a variety of AI-based tools have been widely integrated into clinical workflows for both HCC screening and diagnostic characterization, with certain technologies demonstrating potential applicability across both domains (21, 22). Nevertheless, clearly delineating the intended application scenario of each model remains crucial for guiding its appropriate clinical implementation and validation.

In this study, we reviewed the current studies that describe AI-based models for screening HCC and focused on the applications, challenges and future perspectives of AI in HCC screening. We aimed to comprehensively evaluate the role and limitations of AI in HCC screening to improve early HCC detection rates and patient prognosis.

## 2 AI for HCC screening

**FIGURE 2:**
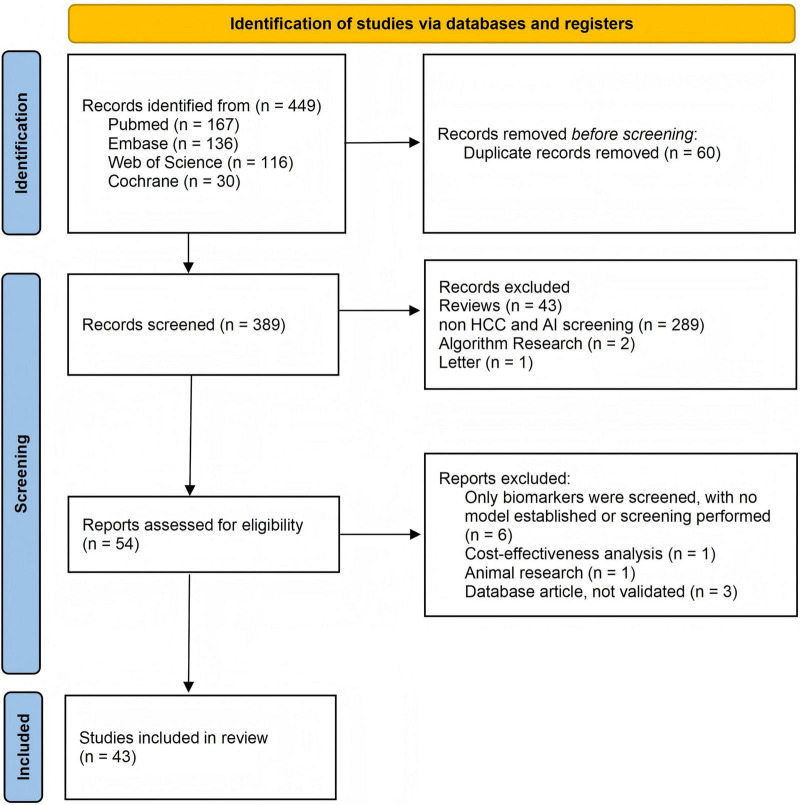
Flowchart of the literature search and study selection.

A comprehensive search of the literature was conducted in English on human subjects via four major databases: PubMed, Embase, Cochrane and Web of Science. The search aimed to identify relevant articles published from inception until November 14th, 2025. The search strategy employed a combination of the following key terms and their variations: “hepatocellular carcinoma,” “hepatic carcinoma,” “HCC,” “artificial intelligence,” “machine intelligence,” “computational intelligence,” “cancer screening,” “cancer early detection,” and “early diagnosis of cancer.” The initial search identified 449 papers: 167 from PubMed, 136 from Embase, 30 from the Cochrane Library and 116 from the Web of Science. After excluding duplications, 389 article titles and abstracts were analyzed. The full texts of 54 articles, which were relevant only to AI in HCC screening, were assessed for eligibility. Finally, 43 articles that met our inclusion criteria were included in this review (Figure 2). A summary of these studies is shown in Supplementary Table 1.

### 2.1 Serum biomarkers

Serum biomarker testing offers a non-invasive and readily implementable approach. Its feasibility and effectiveness for the early detection of HCC are well established (23). Alpha-fetoprotein (AFP) remains the most widely used serological marker for HCC screening. It typically stabilizes in normal adults by 8–12 months of age and is elevated in patients with liver disease, particularly primary liver tumors (hepatoblastoma in children and HCC in adults) (24). However, its sensitivity and specificity are suboptimal, particularly for early-stage HCC detection (25). The latest meta-analysis revealed the diagnostic performance of AFP for early HCC detection, with a sensitivity of only 49.1%, despite an 87.9% specificity (26). The sensitivity of AFP for HCC detection may be influenced by the underlying etiology of HCC. For example, patients with HCC associated with hepatitis B virus (HBV) infection typically present higher serum AFP levels than those with HCC caused by other factors (27). The low sensitivity of AFP diminishes its effectiveness in the early detection of HCC, thereby limiting the utility of serum biomarkers in HCC screening.

AI has become instrumental in bioinformatics, especially in managing large-scale datasets. Numerous studies have utilized machine learning algorithms combined with data from repositories such as the Gene Expression Omnibus (GEO) and the Cancer Genome Atlas (TCGA) to identify feature genes, resulting in the discovery of multiple clinically relevant biomarkers and improving the accuracy and efficiency of HCC screening (28–31). One study leveraged the flexibility of bioinformatic approaches by combining AI and bioinformatics to explore the relationship between diabetic kidney disease and HCC and to identify associated diagnostic markers (32). The integration of AI and bioinformatics enables more precise identification of typical genes associated with HCC of unusual etiology, thereby contributing to the advancement of precision screening and personalized therapeutic strategies.

Cancer-associated autoantibodies (AAbs) can arise early in carcinogenesis and are triggered by the expression of tumor-associated antigens in premalignant or malignant tissues. The ability of the immune system to amplify and sustain specific immune responses makes AAbs promising biomarkers for early cancer detection. Zhang et al. (33) employed human proteome microarrays and an ANN to identify a 7-autoantibody panel for HCC detection. This panel demonstrated superior performance to AFP, particularly in AFP-negative and early-stage (BCLC 0/A) HCC patients. Sato et al. (34) established a novel machine learning-based predictive model for HCC. Their framework employed a grid-search method to optimize classifiers and their hyperparameters, achieving a predictive accuracy of 87.34% and an AUC of 0.94. This performance was superior to that of the standard serum biomarkers AFP, des-gamma-carboxyprothrombin (DCP), and AFP-L3.

AI also aids in the early detection of HCC in patients with diverse characteristics. In patients with HCV-related cirrhosis, the incidence of HCC has decreased markedly among those who achieve a sustained virologic response (SVR) (35, 36). Traditional scoring systems based on routine clinical features overlook population heterogeneity or predictors related to viral eradication (37). Audureau et al. (38) developed novel machine learning-based prognostic models to predict the risk of HCC in patients with compensated HCV-related cirrhosis according to virological status. The key predictors differed between groups: in non-SVR patients, the platelet count and the γ-glutamyl transpeptidas (GGT), AFP, and albumin levels were the most influential, whereas in SVR patients, the prothrombin time, Alanine Aminotransferase (ALT) level, age, and platelet count were predominant. These predictors stratify patients into high- and low-risk populations, facilitating triage for modality-appropriate screening and thereby optimizing the effectiveness and cost efficacy of HCC surveillance. Furthermore, HCC is a major complication of hereditary tyrosinemia type 1 (HT-1), a rare inborn error of metabolism. Since AFP has limited sensitivity for early-stage HCC, Fuenzalida et al. (39) applied a machine learning approach to multidimensional data from HT-1 cohorts. They identified ALT as a promising biomarker that may precede AFP elevation, suggesting a cutoff of 29 U/L to improve the specificity of early HCC detection.

These AI-discovered biomarkers aid in early HCC detection, either directly through risk stratification or by refining AFP-based screening, collectively improving diagnostic sensitivity and specificity to inform early treatment strategies.

### 2.2 Digital biomarkers

Recent advances in next-generation AI, particularly deep neural network (DNN) algorithms, have significantly greatly enhanced biopattern recognition (40). The identification of digital biomarkers via serum surface-enhanced Raman spectroscopy (SERS) has positioned antibody-free SERS detection as a promising tool for cancer screening. Cheng et al. (41) first developed a nanoplasmonic biosensing chip (NBC) for SERS-based, one-drop blood tests. Next, they integrated DNN modeling with this platform to define a digital HCC biomarker, achieving automated cancer identification within minutes with >92% accuracy and AUC values >0.93 (42).

Urine is well suited for large-scale screening owing to its non-invasive collection, cost effectiveness, and ease of storage (43). As the primary organ for amino acid metabolism, liver damage induces intracellular metabolic dysregulation, consequently increasing serum amino acid concentrations and their loss in urine (44, 45). Alterations in the urinary levels of porphyrin derivatives and bilirubin were observed in patients with HCC. Furthermore, 2,3-Dinor-6-keto-PGF1α, the primary urinary metabolite of systemic prostacyclin, is elevated in cirrhosis. Phosphocreatine and N-glycolylneuraminic acid are upregulated during HCC progression. Urine testing may provide a convenient and rapid method for HCC screening. Dawuti et al. (46) demonstrated that the diagnostic sensitivity for HCC was significantly greater when urinary SERS spectra were analyzed with a support vector machine (SVM) algorithm than when serum AFP was used (85.5% vs. 34.5%). Dou et al. (47) also reported that the combination of urine fluorescence spectroscopy and the SVM algorithm has great potential for non-invasive screening of HCC (diagnostic accuracy was 83.42%, sensitivity was 93.55%, and specificity was 88.00%). Shi et al. (48) developed multiscale trimetal oxide heterojunctions for high-quality urinary metabolic fingerprinting. By employing a machine learning algorithm and mass spectrometry, the platform was trained to identify HCC with an accuracy of 93.3% and was also effective in detecting early-stage, AFP-negative HCC.

Digital biomarkers can also be extracted from medical images. Currently, such imaging-based digital biomarkers are employed primarily for predicting treatment efficacy and facilitating therapy stratification (49–51). However, a recent study utilized AI techniques to develop an HCC prediction model for patients with chronic hepatitis B on the basis of CT-derived digital biomarkers (52). This model demonstrated superior performance compared with conventional models relying solely on clinical and demographic data, thereby offering a novel approach to personalized monitoring and screening strategies. Integrated models that combine imaging features with clinical and demographic data may offer a viable strategy for enabling both large-scale screening and refined risk stratification. Current studies have explored the use of ultrasonic biomarkers for the assessment of Achilles tendon integrity and the prediction of breast cancer recurrence (53, 54). Further research on digital biomarkers related to HCC is anticipated.

### 2.3 Liquid biopsy

Liquid biopsy primarily comprises circulating tumor cells (CTCs), cell-free DNA (cfDNA), and extracellular vesicles (EVs). As a minimally invasive procedure utilizing readily accessible biofluids such as blood or urine, it offers significant advantages over tissue biopsy for HCC screening. However, each component faces distinct technical hurdles. CTCs, which are tumor-derived cells in the circulation, are exceedingly rare (approximately one cell per 10^6^–10^7^ leukocytes), and their concentration correlates with disease burden, rendering them suboptimal for early-stage detection (55). Similarly, the tumor-derived fraction of cfDNA, known as ctDNA, often constitutes less than 1% of total cfDNA, with levels decreasing to approximately 0.01% in early-stage cancers, posing a paramount challenge for analytical sensitivity (56). Moreover, EV-based applications are confounded by insufficient tissue specificity and purity, complicating the discrimination of tumor-derived EVs from their non-malignant counterparts (57). Consequently, these limitations have impeded their broad clinical translation. Among these, circulating nucleic acids, especially cfDNA, have shown considerable promise. Recent studies have indicated that tumor-specific genetic alterations in ctDNA can achieve superior sensitivity and specificity over AFP for diagnosing early-stage HCC (58, 59).

AI plays a pivotal role in enhancing diagnostic accuracy by enabling the identification of these tumor-specific genetic alterations. For example, Tao et al. (60) employed machine learning to develop a model based on somatic copy number aberrations for early HCC detection in HBV patients, which achieved impressive AUCs of 0.920 and 0.812 in two independent validation cohorts. Similarly, Samir et al. (61) leveraged machine learning to predict HCC by integrating long non-coding RNA (lncRNA) expression profiles with conventional laboratory biomarkers, attaining 100% sensitivity and 97% specificity. Furthermore, Yu et al. (62) identified nine recurrent fusion genes in HCC and developed a logistic regression model that combined two of these fusions with serum AFP levels, achieving 94.8% accuracy in the training cohort. These studies collectively underscore the transformative potential of AI-driven approaches in refining HCC diagnostics.

However, liquid biopsy still faces several challenges in the screening of HCC. Clonal hematopoiesis-related mutations may confound the identification of tumor-derived mutations in ctDNA analyses, necessitating careful bioinformatic filtering (63). The low fractional abundance of ctDNA in early-stage HCC poses significant technical challenges, as it approaches the detection limits of conventional polymerase chain reaction, making it difficult to distinguish true somatic variants from technical artifacts (64). Moreover, current research efforts remain largely confined to a single class of liquid biopsy biomarkers. Future studies should prioritize the integrated analysis of multiple biomarker classes from a single blood draw to unlock more comprehensive diagnostic information. Current research efforts integrating liquid biopsy and AI are predominantly concentrated on the domain of precise diagnosis, with a primary focus on enhancing specificity to reduce false-positive rates. In contrast, AI-driven models utilizing liquid biopsy for HCC screening must prioritize higher sensitivity to minimize false-negative results and prevent disease progression.

### 2.4 Ultrasound

Ultrasound is the most commonly used modality for HCC screening and detection, offering the advantages of being non-invasive and free from radiation exposure (65). According to the European Association for the Study of the Liver (EASL) guidelines, ultrasound is the recommended examination for HCC screening in all high-risk populations every 6 months (6). This recommendation is consistent with the American Association for the Study of Liver Diseases (AASLD) and Chinese guidelines (66, 67). It has high sensitivity for detecting liver space-occupying lesions >2 cm, but its sensitivity and specificity for early-stage HCC detection remain unsatisfactory (68). Furthermore, the diagnostic performance of ultrasound is significantly operator dependent and can be adversely affected by factors such as ascites and obesity (69).

Recent advances in AI technologies have substantially improved the diagnostic accuracy of ultrasound for HCC detection (70). Two retrospective studies developed AI-based systems for HCC identification among FLLs in ultrasound images, each demonstrating >80% diagnostic accuracy (20, 71). AI also significantly enhanced the diagnostic accuracy of non-expert operators for HCC. A randomized controlled trial demonstrated that non-expert operators using AI-assisted ultrasound not only achieved a significantly higher detection rate of HCC among FLLs but also maintained a comparable rate of false-positive findings relative to conventional ultrasound (72). Furthermore, since serum AFP levels are elevated in only one-third of HCC patients, AI plays a crucial role in assisting ultrasound in identifying biomarker-negative HCC. Zhang et al. (73) developed a deep learning model capable of identifying AFP-negative HCC among FLLs in high-risk patients, achieving an AUC of 93.68%. Contrast-enhanced ultrasound (CEUS) serves as a key diagnostic imaging modality for HCC, advancing conventional ultrasound from screening to definitive diagnosis. While it has 80%–94% sensitivity for early HCC and 63%–70% sensitivity for small lesions (≤2 cm), its performance remains operator dependent and susceptible to interpreter fatigue (74). AI enables rapid analysis and quantification of large datasets, accurate identification of subtle patterns and high-throughput diagnosis without fatigue (71). Ding et al. (75) developed a CEUS-based AI model for multiclass classification of FLLs, which demonstrated superior performance to that of junior radiologists and comparable accuracy to that of senior CEUS specialists. Feng et al. (76) introduced a neural network model that extracts perfusion features through a multiview learning process to differentiate HCC from other malignant conditions, showing strong potential for application in CEUS. Although CEUS is primarily utilized in the diagnostic evaluation of HCC, its high sensitivity for small lesions (≤2 cm) and early-stage HCC may offer potential utility in screening specific populations.

The integration of AI into ultrasound is limited by the inherently dynamic nature of image acquisition and interpretation. Early studies by Shmauch et al. (77) and Yamakawa et al. (78) demonstrated feasibility but were limited by imprecise lesion localization or required manual input, falling short of full automation. A significant advancement was achieved by Tiyarattanachai et al. (79), Xu et al. (80), and Rhyou et al. (81), who utilized video data and object detection models such as YOLOv5 to develop systems capable of fully automated interpretation, outperforming human operators in sensitivity and specificity. While these studies focus on diagnostic accuracy, detection speed—critical for capturing transient anatomical details in real-time scanning—remains underexplored. Furthermore, current automation is largely confined to image analysis; the physical scanning process still relies on human operators. Future research must pursue end-to-end automation, integrating AI-driven robotic scanning. Such a system would drastically reduce costs, increase accessibility in remote areas, and improve early detection rates. To ensure reliability, AI interpretation should be coupled with a secondary verification AI model, with human arbitration for discrepant results, establishing a robust, supervised screening workflow. In cases of discrepancy, human intervention is required for further identification. Given the success of fully automated ultrasound systems, we anticipate the development and validation of fully automated CEUS.

### 2.5 CT

While contrast-enhanced CT is predominantly considered a diagnostic modality, its evolving role within the HCC screening-to-diagnosis continuum is increasingly recognized as crucial. In a comprehensive HCC screening program, CT fulfills two critical functions: (1) serving as a secondary or confirmatory screening tool for high-risk individuals with suboptimal ultrasonography results or elevated clinical suspicion (82, 83), and (2) providing a rapid triage mechanism for the characterization of indeterminate nodules identified during initial ultrasound surveillance (82). The integration of AI into CT workflows substantially enhances the feasibility and accuracy of its screening-related applications, potentially reducing time-to-diagnosis and optimizing resource utilization. Studies indicate that deep learning models utilizing multiphase contrast-enhanced CT and clinical data can effectively extract and integrate radiomic features relevant to diagnosis, achieving diagnostic performance comparable to expert consensus. When deployed as an assistive tool, these models have been shown to improve clinician sensitivity in detecting tumors such as ICC (82). Furthermore, deep learning algorithms trained on contrast-enhanced CT images have demonstrated success in predicting microvascular invasion and histopathological grade in HCC, enabling non-invasive assessment of tumor biology (83). Therefore, CT not only remains a cornerstone for diagnosis and treatment response prediction but, through AI integration, also holds significant promise for screening and early risk stratification.

The sensitivity of CT alone for detecting HCC lesions is 65%, which decreases to 40% for lesions <2 cm (84). By automatically capturing high-level imaging features, AI enhances the diagnostic accuracy for HCC and streamlines the diagnostic workflow (85). Lysdahlgaard et al. (86) employed machine learning algorithms to analyze and classify radiomic features derived from both hepatic tumors and normal liver tissue in a limited CT dataset, achieving classification accuracies ranging from 94% to 100%. Şahin et al. (87) developed a deep learning model using CT images from 122 patients, which achieved exceptional diagnostic accuracy (95.35%) for HCC and significantly outperformed conventional diagnostic methods. Wang et al. (88) employed an AI-based methodology utilizing early CT radiomics features to predict hyperspectral imaging characteristics, enabling non-invasive tumor prediction and early screening with >90% classification accuracy. Owing to overlapping imaging features between small HCC (≤2 cm) and benign cirrhotic nodules, the direct application of deep learning algorithms to multiparametric images for small HCC detection remains challenging. Zheng et al. (89) developed an integrated pattern-matching and deep learning model that achieved a sensitivity of 89.74% and a positive predictive value of 85.00% in small HCC diagnosis.

Recently, AI has also been employed to detect subtle patterns on non-contrast CT scans that are imperceptible to human observers, enabling highly sensitive and specific cancer detection. Cao et al. (90) developed a deep learning model that identified pancreatic cancer on non-contrast CT with 92.9% sensitivity and 99.9% specificity, significantly outperforming radiologists. Similarly, Peng et al. (91) created an AI algorithm for HCC detection via non-contrast CT, achieving an AUC value of 0.789. The development of accurate AI algorithms for HCC detection enables the application of non-contrast CT for opportunistic screening by reducing both radiation exposure and costs. The combination of non-contrast CT and AI plays a vital role in screening special populations, such as patients with contrast agent allergies.

The clinical implementation of AI-assisted CT models faces several limitations, such as independence from high-quality imaging data and heterogeneity in scanning protocols across institutions. Notably, the false-negative rate for detecting HCCs < 1 cm remains substantial. Advancing the detection of subcentimeter HCCs through combined CT and AI approaches represents a critical research priority to reduce interval tumor progression. While CT offers high diagnostic accuracy and remains a cornerstone in HCC diagnosis, its inherent limitations—such as ionizing radiation exposure and considerable cost—preclude its adoption for routine surveillance. Nevertheless, AI-enhanced CT holds distinct, actionable value within a risk-stratified screening paradigm. Specifically, it can serve as (1) a problem-solving modality for indeterminate ultrasound findings, (2) a high-precision screening tool for selected high-risk subgroups, and (3) an opportunistic screening resource when non-contrast CT is performed for other indications. Future screening algorithms may strategically integrate AI-CT at key decision points to enhance the efficiency and accuracy of the screening cascade, thereby transcending the conventional dichotomy between screening and diagnostic imaging.

## 3 Challenges of AI applications in HCC screening

### 3.1 Limited generalizability of the models

A key limitation of AI applications in HCC screening is the poor generalizability of models, primarily due to interpatient variability and the heterogeneous etiologies of HCC. The majority of studies have been performed in patients with chronic viral hepatitis B. However, metabolic-associated liver disease has become the most common cause of liver disease worldwide and is the leading cause of non-cirrhotic HCC, with approximately 25%–30% of patients developing HCC in this context (92, 93). This change has not only challenged the diagnostic capabilities of AI models, as previously validated radiographic systems are not applicable in this different cohort, but has also challenged HCC screening and surveillance practices. Furthermore, overfitting is also a main reason why a model demonstrates high accuracy on the training data but fails to generalize to unseen test datasets (94). This occurs when the model memorizes dataset-specific noise and patterns that are not generalizable, a situation often exacerbated by limited training data. Moreover, modest sample sizes also increase the risk of overfitting. To mitigate overfitting and enhance the generalization performance, techniques such as input feature reduction and increased regularization can be employed (Dropout: a simple way to prevent neural networks from overfitting). AI models from academic prototypes to clinical implementation need to be as rigorously assessed.

### 3.2 Ethical challenges

The key ethical issues central to ongoing ethical debates encompass data privacy and security, the assignment of moral accountability and liability for AI errors, and the mitigation of inherent biases and algorithmic inequalities within AI-driven HCC screening.

A trusted data space is critically important for ensuring privacy, data security, and responsible data processing and storage. The establishment of such infrastructure not only accelerates the flow of data among data providers, service providers, and data users in AI applications but also ensures privacy and security in data usage. The National Data Administration of China has issued the Trusted Data Space Development Action Plan (2024–2028) (95), which outlines a structured framework to promote the development of trusted data spaces at the corporate, industrial, municipal, individual, and cross-border levels. This vision aligns closely with the AI-based HCC screening and surveillance strategy proposed in our work. By leveraging trusted data space platforms, it becomes feasible to develop dedicated AI systems for disease screening and monitoring. Primary care institutions can act as both data providers and end-users. Municipal and international data spaces can facilitate secure data transmission and storage while also supporting the training, maintenance, updating, and oversight of AI models. This infrastructure provides a robust foundation for scalable and ethically aligned AI-powered disease screening. Additionally, cancer diagnosis often involves complex ethical issues. Whether to disclose the diagnosis to the patient is a critical consideration in clinical practice, particularly for instant testing modalities such as ultrasound examinations. It is recommended that AI systems use ambiguous terms—such as “nodule” or “space-occupying lesion”—when interpreting reports and advise further hospital-based diagnostic assessment to avoid potential ethical complications.

The ethical implications, accountability, and attribution of responsibility in cases of errors committed by AI systems should be considered. Compared with reactions to comparable human errors, public responses to errors by AI systems tend to be more pronounced. Moreover, failures in a single AI system are often overgeneralized to imply systemic deficiencies across all AI technologies. As a result, the ethical consequences and erosion of trust attributable to AI errors can be substantially amplified (96). In light of these challenges, establishing a clear chain of accountability for AI and robotic systems, with explicitly defined roles and responsibilities for manufacturers, healthcare institutions, and medical practitioners, is essential. An incident reporting mechanism should be implemented to enable healthcare providers to promptly document errors and adverse events related to AI applications.

Bias in AI systems largely originates from the underlying data used to train these models (97, 98). Most current AI tools are developed on the basis of historical institutional datasets, which makes them prone to inheriting biases resulting from imbalanced representations of different demographic groups in disease datasets (99). For example, an AI tool designed to predict healthcare costs, when trained primarily on a White population, may perform poorly when applied to African American patients (100). Regional and ethnic heterogeneity in HCC risk factors introduces significant complexity to the development and validation of AI models for screening, challenging their performance stability and fairness across diverse populations. Globally, epidemiological patterns vary substantially. Asia bears approximately 70% of the worldwide HCC burden, with HBV infection representing the predominant etiological factor (101). Similarly, HBV remains the leading cause in Africa (102). In Europe, risk profiles display distinct regional patterns: hepatitis C virus (HCV) is the primary contributor in Western Europe, whereas ALD accounts for nearly half of HCC cases in Central and Eastern Europe. Overall, HBV is responsible for only approximately 16% of European cases (103, 104).

Therefore, rather than adopting a one-size-fits-all general model, a more refined strategy—developing population-specific AI models—may more effectively mitigate bias while preserving important biological differences. This approach entails well-annotated data collection, rigorous data curation and monitoring during the data acquisition phase, as well as the implementation of tailored modeling strategies and clear recommendations regarding the scope of application during the model design and deployment stages.

Disparities in the number of AI models across countries with different income levels contribute to AI inequality (105). Typically, only researchers in well-resourced settings are able to generate or collect sufficient data and implement novel AI methodologies (106, 107). Algorithms trained on data from high-income countries are deployed in real-world contexts in low- and middle-income countries (LMICs). However, without adequate consideration of the unique local conditions and needs of LMICs, these systems may lack generalizability and broad effectiveness (108, 109). Moreover, infrastructural limitations in LMICs restrict the applicability of AI models, further exacerbating AI inequality. Recent empirical evidence indicates that variations in technical parameters significantly influence the performance of AI algorithms developed from medical imaging data (110–113). For example, AI models developed on the basis of 3T MRI equipment may not be suitable for many African nations and other LMICs, where scanners typically operate at ≤1.5 T. Finally, differences in available data modalities across income settings also contribute to AI inequality. To promote more equitable AI, findings from Jenny Yang et al. (114) were obtained suggest that transfer learning and fine-tuning models with LMIC data can enhance the generalization capabilities of AI systems. Nonetheless, the study emphasizes the importance of developing resilient AI tools specifically designed for healthcare systems in LMICs. Therefore, greater attention should be given to creating algorithms compatible with more accessible data types and increasing the number of AI models trained on data from LMICs, thereby improving the applicability and relevance of AI technologies in these settings (115).

## 4 Translational feasibility and implementation pathways

Based on the synthesized evidence, we evaluate the translational readiness of various AI applications and their enabling infrastructures for HCC screening, focusing on technical validation, integration potential, and preliminary implementation evidence.

### 4.1 Most promising for near-term adoption: AI-assisted ultrasound detection and risk stratification tools

AI-assisted ultrasound: This approach constitutes the most direct and pragmatically viable pathway for near-term integration. Substantial evidence indicates that AI can significantly enhance the sensitivity of HCC detection by both expert and non-expert operators during real-time or retrospective image analysis (71, 72). Its principal advantages include the following: (1) Workflow compatibility augments the established first-line screening standard (biannual ultrasound) without necessitating fundamental changes to the clinical care pathway. (2) Targeting a critical gap: It directly addresses the well-recognized operator-dependent variability of ultrasonography, a major impediment to screening effectiveness. (3) Phased deployment: Implementation can commence with AI serving as a “second reader” or decision-support system, thereby minimizing workflow disruption and facilitating clinical adoption and trust.

Clinical data-driven AI risk stratification models: These models integrate readily available clinical and laboratory variables to calculate individual HCC risk, representing a highly implementable tool (38). Their key strengths are as follows: (1) Low incremental resource burden: They leverage data routinely captured within electronic health records. (2) Actionable clinical output: They enable personalized screening strategies, such as intensifying surveillance for high-risk patients or safely extending intervals for low-risk individuals, thereby optimizing resource allocation. (3) Streamlined regulatory pathway: Classified as Software as a Medical Device (SaMD) that does not involve novel hardware or diagnostic procedures, these models may navigate a relatively more defined regulatory approval process.

### 4.2 Promising but with specific hurdles: integrated serum biomarker panels, CT-based radiomics and trusted data spaces

AI-discovered/integrated serum biomarker panels: Panels such as the 7-autoantibody assay or models combining AFP with other markers have demonstrated superior diagnostic performance compared to AFP alone (33, 34). Their feasibility hinges on: (1) Standardization and regulatory approval: Transitioning from research-based assays to standardized, FDA/CE-approved *in vitro* diagnostics represents a critical development step. (2) Demonstration of clinical utility: Prospective trials are needed to confirm that their application improves early detection rates and patient-relevant outcomes compared to current surveillance standards.

CT-based radiomics/AI models: Although these models achieve high diagnostic accuracy, their application in population-wide screening is constrained by the inherent limitations of CT (86, 87). Their near-term implementation is more feasible in targeted contexts, such as surveillance of subpopulations with indeterminate nodules or as a triage tool following inconclusive ultrasound findings.

Trusted data spaces: The establishment of secure, interoperable, and ethically governed data platforms is a foundational enabler for advanced AI development. While not a direct screening tool, creating such trusted data spaces at institutional or regional levels is a tangible near-to-mid-term objective but faces distinct non-technical challenges. These include defining data sovereignty and access frameworks, ensuring compliance across legal jurisdictions, and establishing viable governance and sustainability models. Although pilot projects in related fields have demonstrated technical feasibility—and initiatives such as China’s plan to establish over 100 trusted data spaces by 2028 aim to provide solutions and best practices—their broad implementation for HCC screening will require coordinated policy alignment and dedicated investment.

### 4.3 Future horizons: liquid biopsy with AI, fully automated ultrasound systems, and global AI-integrated surveillance ecosystems

AI-enhanced liquid biopsy (cfDNA/EVs): Despite promising proof-of-concept studies (60–62), detecting the low circulating tumor DNA fraction in early-stage HCC remains technically challenging. Broader clinical adoption awaits the development of more robust, cost-effective, and standardized detection platforms.

A mid- to long-term objective: While conceptually promising for expanding screening access, such systems require substantial advances in robotic scanning technology and real-time AI interpretation of dynamic image data. Their development and rigorous clinical validation represent a mid- to long-term objective.

Global AI-integrated surveillance ecosystems: The vision of a globally interoperable AI-driven surveillance network represents a long-term, transformative goal. Its realization depends on the maturation and integration of multiple components: validated AI tools, secure and scalable data infrastructures, and automated screening technologies. Key challenges include ensuring technical interoperability across diverse AI systems, harmonizing international clinical and regulatory standards, and addressing global equity to ensure accessibility in low-resource settings. Progress will depend less on technological feasibility and more on sustained international collaboration, governance alignment, and political commitment.

The integration of AI into HCC screening is expected to follow a staged pathway. In the near term, prioritizing the implementation of AI-augmented ultrasound and clinical risk stratification tools within existing workflows offers the most pragmatic and impactful strategy. Success in these initial applications will establish essential clinical trust and infrastructural foundations to support the future adoption of more advanced, multimodal AI systems.

## 5 Future perspectives

### 5.1 Risk-stratified HCC screening

The developmental pathogenesis of HCC remains incompletely understood and exhibits pronounced heterogeneity across ethnicities and etiologies. Current screening paradigms, which predominantly target broad populations, may lack the precision offered by risk-stratified strategies capable of optimizing early detection in high-risk subgroups. Indeed, the risk of HCC is not uniform; it is substantially elevated in patients with cirrhosis, who face an annual incidence of 2%–4% (116). Consequently, both the AASLD and EASL guidelines endorse routine surveillance for this demographic, an approach proven to improve overall survival (117). With the evolving landscape of HCC etiology, MASLD and alcohol-associated liver disease are projected to become major sources of HCC in the future. The large population base and relatively low incidence rate associated with these conditions impose a substantial burden and create significant barriers to effective screening and surveillance. Consequently, there is a pressing need to develop and validate AI-assisted diagnostic models for HCC that are applicable across diverse clinical scenarios. Risk-stratified models tailored to distinct high-risk populations should be implemented. For individuals at lower risk levels, fully automated end–end ultrasound should serve as the foundational screening and surveillance modality, supplemented by clinical features such as age, sex, and liver disease status, to guide specific screening strategies. As risk levels increase, the protocol should selectively incorporate additional tests—such as serum biomarkers, liquid biopsy, gut microbiome analysis, CT, and MRI—based on patient stratification to increase diagnostic accuracy. For individuals at intermediate to high risk (e.g., cirrhosis with prior indeterminate lesions), screening may commence with or quickly escalate to AI-assisted CT, balancing the higher sensitivity for small lesions against the increased cost and radiation exposure. This targeted approach maximizes the value of CT within a screening context. This stratified approach is expected to significantly improve screening efficiency, enhance cost-effectiveness, optimize the allocation of finite healthcare resources, and ultimately improve HCC outcomes.

**FIGURE 3:**
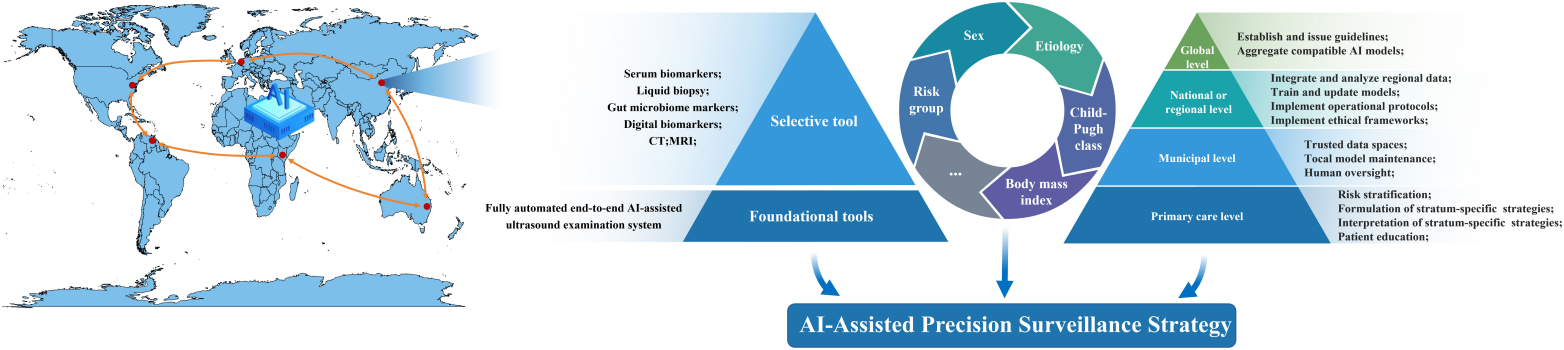
Multi-tiered architecture for implementing an AI-assisted precision surveillance strategy. The schematic depicts a hierarchical framework spanning from primary care to global levels. At the primary care level, AI supports risk stratification, strategy formulation, and patient education. Municipal levels host trusted data spaces for system maintenance and oversight. National/regional platforms are responsible for data integration, model training, and implementing context-specific protocols. A global tier issues guidelines and aggregates compatible AI models to enhance generalizability, collectively enabling a scalable and ethically governed precision screening ecosystem. CT, computed tomography; MRI, magnetic resonance imaging; AI, artificial intelligence.

The establishment of an AI-based precision screening system for HCC is proposed (Figure 3). From an organizational perspective, a multitiered AI infrastructure spanning primary care and municipal, national, and global levels should be constructed. At the primary care level, AI should assist healthcare providers in risk stratification, formulation and interpretation of stratum-specific screening and surveillance strategies, and patient education. At the municipal level, trusted data spaces should be established and expanded to host AI-assisted screening and surveillance systems, which are responsible for local model maintenance, human oversight, and data storage. At the national or regional level, AI-based platforms should be developed to integrate and analyze regional data, train and update models, and implement operational protocols and ethical frameworks tailored to local contexts. At the global level, an international platform should be established to issue guidelines, aggregate compatible AI models, and enhance generalizability across populations.

### 5.2 Accessible HCC screening

The majority of patients do not adhere to recommended HCC screening strategies. Davila et al. (118) reported that among patients diagnosed with HCC, no more than 28% had undergone at least one ultrasound examination within the 3 years preceding diagnosis. The same research group, in a study involving a larger at-risk population, also confirmed low surveillance rates, with only 12% and 59% of patients receiving consistent and inconsistent surveillance, respectively. Both studies were conducted in a developed country, and the situation is likely more severe in developing or resource-limited regions (119). This may partly explain the current low rate of early HCC diagnosis, underscoring the urgent need for improved screening strategies. Some studies have employed methods such as mailed outreach and primary care-based clinical reminders to increase the degree of participation in HCC screening, with positive outcomes reported (120, 121). However, these approaches are not cost effective and rely heavily on patient health literacy, making them difficult to implement in underserved areas.

Therefore, we propose the development of a fully automated end-to-end ultrasound examination system. This strategy would involve primary healthcare providers or community health workers as the main actors in HCC screening, implementing a grid-based approach to cover at-risk populations within their jurisdictions. Using ultrasound as the foundational modality, these primary care personnel—who have the most frequent contact with high-risk groups—can conduct screenings more efficiently than specialists, whose coverage is often broader but less focused. The implementation of a fully automated ultrasound process would significantly reduce technical personnel costs, allow flexible scheduling for patients, and greatly decrease the cost per screening session. Consequently, the primary role of grassroots healthcare workers would shift to reminding and supervising the effective execution of HCC screening, thereby alleviating their workload. This approach is expected to not only increase the coverage of HCC screening but also reduce the overall cost of screening.

### 5.3 Cost-effective HCC screening

Cost-effectiveness is a critical determinant for the practical application of AI in clinical settings. Multiple studies have demonstrated that screening and surveillance for HCC in high-risk populations, particularly among patients with cirrhosis, is cost-effective (122–124). However, other research has indicated that in countries with a low incidence of HCC, such screening may not be cost-effective (125). In contrast to risk-stratified monitoring, Kao et al. demonstrated that a precision surveillance strategy represents the most cost-effective approach to HCC monitoring. This strategy integrates four patient-specific factors within risk-defined cohorts and evaluates four potential surveillance protocols, thereby providing a framework for individualized monitoring. Future surveillance stratification and strategies should adopt greater flexibility to accommodate diverse population characteristics. Furthermore, the integration of AI into HCC screening is anticipated to increase cost-effectiveness (126).

## 6 Conclusion

AI technologies are transforming HCC screening paradigms. These innovative approaches leverage comprehensive data mining from medical records, either as stand-alone tools or in combination with conventional serological markers and imaging modalities, to achieve superior diagnostic sensitivity and specificity, significantly reducing the technical barriers associated with operator dependency and image interpretation variability. This synergy may establish real-time AI-assisted HCC screening as a groundbreaking paradigm in early cancer detection. These advancements fundamentally enhance HCC management while aligning with global health priorities to reduce cancer mortality in resource-limited settings. However, owing to the current limitations of AI models in HCC screening due to their poor generalizability and ethical issues, the clinical implementation of AI models poses substantial challenges. Before AI-assisted HCC detection can be incorporated into routine screening protocols, it must undergo rigorous validation in large-scale, high-quality multicenter studies. Furthermore, a critical resolution of the accompanying ethical dilemmas is imperative.
